# Lack of Association between *LOXL1* Variants and Pigment Dispersion Syndrome/Pigmentary Glaucoma: A Meta-Analysis

**DOI:** 10.3390/genes15020161

**Published:** 2024-01-26

**Authors:** Shisong Rong, Xinting Yu

**Affiliations:** 1 Department of Ophthalmology, Massachusetts Eye and Ear, Mass General Brigham, Harvard Medical School, Boston, MA 02114, USA; 2 Department of Medicine, Brigham and Women’s Hospital, Mass General Brigham, Harvard Medical School, Boston, MA 02115, USA; xinting.yu@bwh.harvard.edu

**Keywords:** pigment dispersion syndrome, pigmentary glaucoma, genome-wide association study, genetic associations, systematic review, meta-analysis, animal models

## Abstract

The phenotypic similarities between exfoliation syndrome (XFS)/exfoliation glaucoma (XFG) and pigment dispersion syndrome (PDS)/pigmentary glaucoma (PG), particularly their association with material deposition in the eye’s anterior segment, have prompted investigations into genetic commonalities. This study focuses on the *LOXL1* gene, conducting a comprehensive meta-analysis of three candidate gene association studies. We analyzed three single nucleotide polymorphisms (SNPs) of *LOXL1*: rs1048661, rs3825942, and rs2165241. Our results reveal nominal significance for the exonic SNPs rs1048661 and rs3825942 (*p* ≤ 0.01), but show no significant association for the intronic SNP rs2165241 (*p* = 0.83) with PDS/PG. There was homogeneity across study cohorts (I^2^ = 0), and sensitivity analyses and funnel plots confirmed a lower likelihood of bias in our findings. The lack of a statistically significant association between *LOXL1* variants and PDS/PG at *p* < 0.05 was attributable to the insufficient statistical power of the pooled data, which ranged from 5% to 37% for the three SNPs. This study suggests no association between *LOXL1* variants and PDS/PG. Further validation and exploration of XFS/XFG-associated genes in larger and more diverse cohorts would be helpful to determine the genetic correlation or distinctiveness between these conditions.

## 1. Introduction

Pigment dispersion syndrome (PDS) is defined by the dispersion of pigment from the iris pigment epithelium and its accumulation on anterior segment structures such as the cornea and trabecular meshwork [1]. Among the significant manifestations in PDS patients are iris transillumination defects (ITD), Krukenberg’s spindle, pigmented trabecular meshwork (TM), and retinal lattice degeneration of the retina. In certain instances, the accumulated pigment may lead to alterations in the TM, thus obstructing aqueous humor outflow, resulting in increased intraocular pressure (IOP) and the development of glaucomatous optic neuropathy, eventually leading to the diagnosis of pigmentary glaucoma (PG). The prevalence of PDS is approximately 2.5% in Caucasian populations [2], whereas it is less common in African [3] and Asian populations [4,5]. Pigment dispersion syndrome and PG are recognized as part of a continuous spectrum of the same pathological condition. Studies suggest that 10% to 50% of individuals with PDS will eventually progress to PG [1,6,7,8,9]. In the Western world, PG constitutes approximately 1–1.5% of all diagnosed glaucoma cases [10]. Despite the need for a comprehensive understanding of the disease mechanisms, the precise pathophysiological mechanisms underlying PDS remain incompletely elucidated.

Exfoliation syndrome (XFS) affects anterior segment structures and is characterized by the deposition of white exfoliation material (XFM) on the anterior lens surface and/or pupillary border [11,12]. XFS exhibits a tripartite pattern observable in fully dilated pupils: a central disc, an intermediate clear zone (resulting from iris-induced XFM removal from the lens surface), and a peripheral granular zone. XFM frequently accumulates at the pupillary border, leading to pigment dispersion from the iris into the anterior chamber. This process involves the scraping of XFM by the iris from the lens, causing the rupture of iris pigment epithelial cells and subsequent pigment dispersal. The resulting clinical features include iris sphincter transillumination, loss of the pupillary ruff, TM pigmentation, and pigment deposition on the iris. Exfoliation glaucoma (XFG), a subset of XFS, arises from IOP elevation and is associated with aging [13,14,15]. The prevalence of XFS and XFG exhibits notable similarities with PDS/PG, with a higher prevalence in European descendants, 10% to 20% [16,17,18], and lower prevalence in Middle Eastern [19,20,21], African [22,23], and Chinese individuals [24,25].

Genetic research in the fields of PDS/PG and XFS/XFG has identified various genetic loci and genes associated with these conditions. For PDS/PG, genetic linkage analysis and recent genomics analysis have revealed associations with specific loci and genes, such as 7q35-q36, 18q21, 18q22.1, 2q22.1, *PMEL*, *CPAMD8*, *GSAP*, and *GRM5/TYR*. However, the complete genetic components associated with PDS and PG remain incompletely understood despite these advancements [26,27,28,29,30,31,32,33]. In the case of XFS/XFG, the initial genetic discovery stemmed from a genome-wide association study (GWAS) in 2007, which established a link between XFS/XFG and the *LOXL1* gene. Subsequently, the identification of the *CACNA1A* genetic locus provided further insights into XFS/XFG genetics, reaffirming the *LOXL1* association. A third GWAS uncovered five additional gene loci (*POMP*, *TMEM136*, *AGPAT1*, *RBMS3*, *SEMA6A*) and suggested a protective role of a rare *LOXL1* missense variant against XFS/XFG [34,35,36].

The phenotypic overlap between PDS/PG and XFS/XFG, particularly the higher prevalence in European descendants, pigmentary material deposition in the anterior segment, and iris transillumination, has prompted investigations into a potential genetic link between these conditions. Genetic investigations have primarily focused on the *LOXL1* gene, known for its association with XFS/XFG, in relation to PDS/PG [37,38,39]. Giardina et al. identified significant allele associations in Caucasian patients with PDS/PG for SNP rs2304722, suggesting that certain *LOXL1* haplotypes may modulate the expression of this gene and influence the risk of developing these conditions [39]. Contrastingly, Wolf et al. found no major influence of *LOXL1* polymorphisms on the pathophysiology of PG in a German cohort, although a nonsynonymous polymorphism might predict age at disease onset [38]. Additionally, Rao et al. reported no significant association between the common *LOXL1* SNPs and PDS/PG in their study group, further indicating the specificity of these SNPs to exfoliation syndrome and glaucoma [37]. However, these studies are hindered by inadequate statistical power and have thus yielded inconclusive results. Therefore, it remains to be confirmed whether *LOXL1* variants influence the risk and age of onset of both diseases [37,38,39,40].

To address the limitations posed by small sample sizes in individual studies, this meta-analysis combines existing data to quantitatively assess the genetic association between PDS/PG and XFS/XFG. Additionally, we systematically review the current literature on this topic and outline future directions for genetic research in this area.

## 2. Materials and Methods

### 2.1. Identification of Gene Association Studies Testing

We performed the literature search using Boolean logic, and the search terms incorporated controlled vocabularies (i.e., Medical Subject Heading terms) in the PubMed/MEDLINE databases. The search terms were constructed as follows: ((pigment dispersion syndrome) OR (pigmentary glaucoma)) AND (Medical Genetics OR genotype OR genetics[Subheading] AND genetics) AND (((((lysyl oxidase like 1) OR (LOL)) OR (LOXL)) OR (LOXL1)) OR (CACNA1A OR POMP OR TMEM136 OR AGPAT1 OR RBMS3 OR SEMA6A) (Appendix A). We summarized all the records that met the following criteria: (1) the study tested associations of *LOXL1* variants with PDS or PG; (2) the study population was clearly defined; and (3) the diagnosis of PDS or PG was based on clinical data. We also scanned the reference lists of the research articles, editorials, or reviews identified during the screening process to include all relevant publications. In addition, we also searched the GWAS catalog, UK Biobank, and FinnGen for relevant datasets. The last search was done on 28 November 2023.

### 2.2. Data Extraction

Data extraction was undertaken independently by two investigators (XTY and SSR). Any discrepancies in data interpretation or extraction were resolved by reaching a consensus among all investigators. For studies that did not directly report allele counts, we derived these counts from the available genotype data. In cases where only OR and 95% CI were reported, we calculated the SE using the formula SE = [β − ln(lower limit of 95% CI)]/1.96, where β equals ln(OR) [41]. Results of Hardy–Weinberg equilibrium (HWE) tests from each study were recorded.

### 2.3. Quanlity Assessment and Control of Risk of Bias

To assess the quality of the case-control studies included, we employed the Newcastle–Ottawa Scale (NOS, accessible via http://www.ohri.ca/programs/clinical_epidemiology/oxford.asp, accessed on November 2023), as detailed in Appendix A [42,43,44]. A study scoring six or fewer stars on the NOS was deemed to have poorer quality, therefore, indicating a higher risk of introducing bias [45]. The Newcastle–Ottawa Scale enabled us to gauge the reliability and validity of the included studies.

To evaluate potential publication bias, we constructed funnel plots [46,47,48]. Sensitivity analyses were also performed to verify the robustness of the associations. This involved sequentially excluding studies—those deviating from HWE, or those deemed of suboptimal quality—to assess the impact on the overall results.

### 2.4. Genetic Meta-Analysis

For the meta-analysis, we adhered to published genetic meta-analysis protocol [49,50,51,52,53,54]. We selected single nucleotide polymorphisms (SNPs) and studies based on the following parameters: (1) The studies needed to be original genetic case-control research involving unrelated individuals drawn from defined populations. (2) They had to provide sufficient data for the calculation of odds ratios (OR) and 95% confidence intervals (CI) or standard errors (SE). Exclusion criteria were rigorous, omitting animal studies, case reports, reviews, abstracts, conference proceedings, and editorials to ensure data purity and relevance.

We prioritized the use of data generated from fully balanced case-control comparisons for the meta-analysis when such data were available. To address the issue of duplicate cohorts, we opted for the larger and more recent cohort to avoid redundancy and to ensure the most current data were analyzed.

Meta-analyses were conducted for each SNP reported in two or more cohorts. PDS and PG were grouped as a single phenotype for analysis purposes. We employed an allelic model to assess genetic associations, calculating summary ORs and 95% CIs for each polymorphism using a random-effects model. Heterogeneity among studies was quantified using the I^2^ statistic [55], with an I^2^ value below 25% indicating low heterogeneity. Meta-analyses were performed and relevant plots were generated using Review Manager 5 (RevMan 5). We considered summary *p*-values less than 0.05 as indicative of statistical significance.

We followed the Preferred Reporting Items for Systematic Reviews and Meta-Analyses (PRISMA) guidelines in reporting this systematic review and meta-analysis [56]. We only used published data from relevant studies, thus bypassing the need for a separate Institutional Review Board (IRB) approval for our analysis. IRB approvals were secured for each published study by their respective committees [37,38,39].

### 2.5. Functional Annotation of Gene Variants

To elucidate the functional significance of SNPs associated with the diseases, we utilized in silico functional prediction tools including SIFT [57], PolyPhen [58], CADD [59], and RegulomeDB [60]. Additionally, we analyzed expression quantitative trait loci (eQTL) using the Genotype-Tissue Expression (GTEx) portal, providing insights into how risk alleles might influence nearby gene expression [61].

### 2.6. Power Analysis

The power analysis for the *LOXL1* association study on PDS/PG utilized a two-sided Z-test with pooled variance to assess the statistical power of different group sizes. The analysis compared a case group and a control group with varying sample sizes (e.g., 70 to 90 cases and 100 to 300 controls). It focused on detecting odds ratios ranging from 0.75 to 1.33 for group proportions. In cases, under the null hypothesis (H0), both groups were assumed to have the same allele frequencies (e.g., 0.1 to 0.50), while under the alternative hypothesis (H1), different frequencies were considered (e.g., 0.077 to 0.43). The target significance level was set at 0.05. This methodology helped determine the study’s capability to detect true effects, given its design constraints. We used the R library ‘pwr’ to conduct this analysis.

## 3. Results

### 3.1. Studies Testing XFS/XFG-Associated Genes in PDS/PG

**Figure 1:**
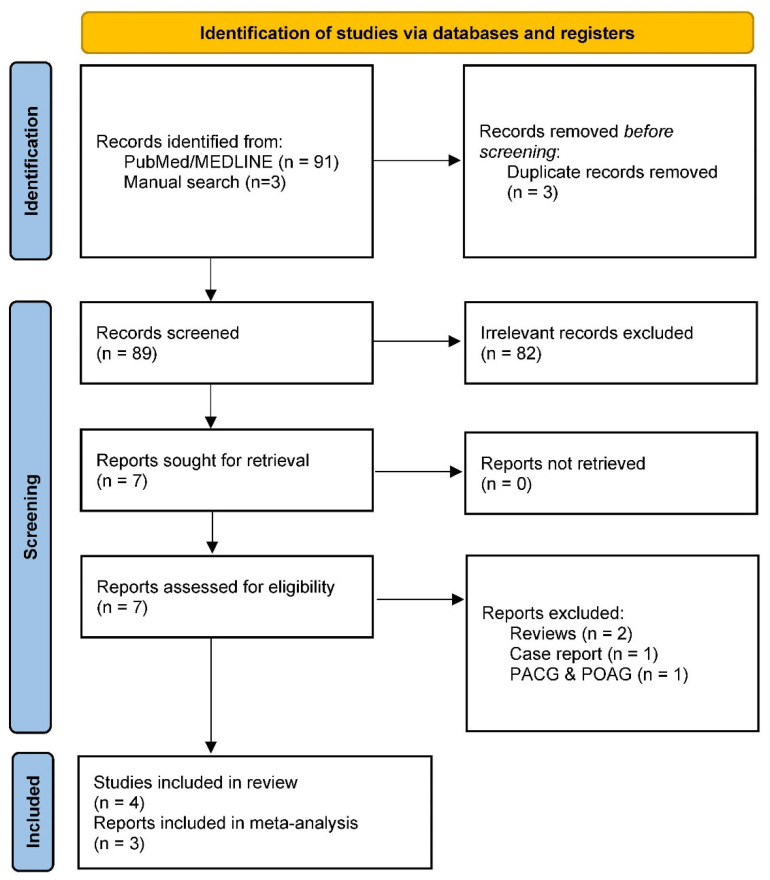
Preferred Reporting Items for Systematic Reviews and Meta-Analyses (PRISMA) 2020 flow diagram for systematic reviews.

Our literature search identified 93 records, of which seven were directly relevant and, thus, selected for in-depth full-text review. Following review, three studies were excluded due to their nature: two were reviews [12,62], one was focused on primary open-angle and angle-closure glaucoma [63], and one was a case report [64] (Figure 1). We did not identify relevant datasets from the GWAS catalog, UK Biobank, and FinnGen.

The remaining three genetic association studies specifically examined 10 *LOXL1* variants in PDS and PG: rs1048661, rs1284049, rs1530169, rs2165241, rs2304722, rs3522, rs3825942, rs750460, rs8818, and rs893818 [37,38,39] (Table 1). Notably, genes traditionally associated with XFS/XFG, such as *CACNA1A*, *POMP*, *TMEM136*, *AGPAT1*, *RBMS3*, and *SEMA6A*, were not examined in PDS/PG. Consequently, our meta-analysis was specifically tailored to assess the implications of the *LOXL1* gene variants in PDS/PG.

### 3.2. Genetic Association of LOXL1 with PDS/PG

To clarify the inconclusive results of individual *LOXL1* genetic association studies, we performed a meta-analysis, summarizing three specific SNPs (rs1048661, rs3825942, and rs2165241) that were examined in at least two studies (Table 1 and Table 2).

**Figure 2:**
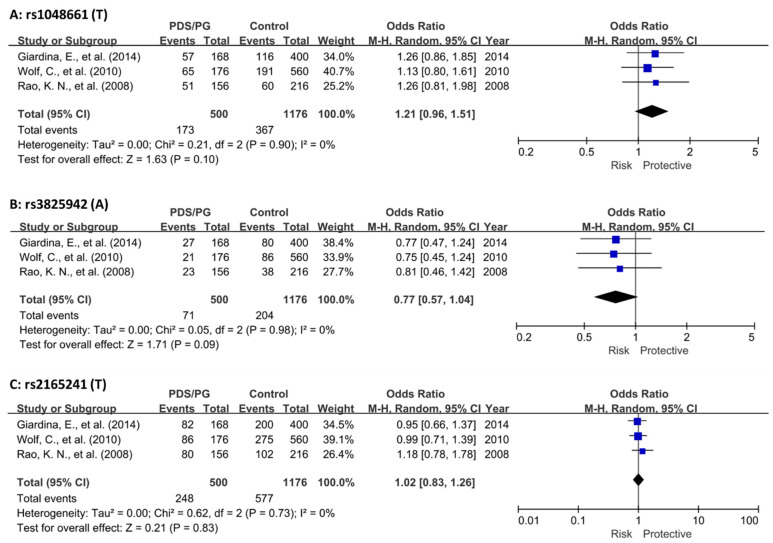
Meta-analysis of *LOXL1* SNPs in combined PDS/PG association studies. (**A**) Meta-analysis of rs1048661 across three cohorts. (**B**) Meta-analysis of rs3825942 across three cohorts. (**C**) Meta-analysis of rs2165241 across three cohorts. In all cases, heterogeneity among the pooled studies was found to be minimal, indicating consistent findings across different cohorts. The diamond shape at the bottom of a forest plot synthesizes all the individual study results into a single, overall estimate. The center of the diamond indicates the combined effect size, and the width of the diamond shows the confidence interval for this combined estimate. CI, confidence interval; PDS, pigment dispersion syndrome; PG, pigmentary glaucoma [37,38,39].

Our meta-analysis revealed that the two exonic SNPs, rs1048661 and rs3825942, exhibited only nominal significance (*p* ≤ 0.01). In contrast, the intronic SNP, rs2165241, showed no significant association (*p* = 0.83) (Figure 2). Notably, the meta-analyses of all cohorts indicated homogeneity (I^2^ = 0). This uniformity likely results from the original studies’ consistent use of Caucasian populations and balanced case-control samples (Figure 2 and Table 2).

### 3.3. Risk of Bias Assessments and Sensitivity Analysis

**Figure 3:**
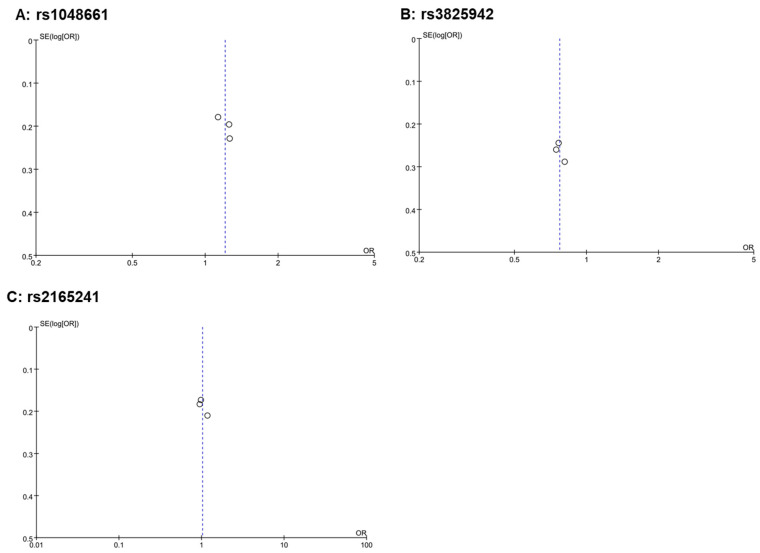
Funnel plots of the three meta-analyses. A funnel plot is a scatter plot used in meta-analyses to detect publication bias. It plots the effect sizes from individual studies on the horizontal axis against a measure of study size or precision (like standard error shown here) on the vertical axis. In the absence of bias, the plot resembles an inverted funnel, with larger, more precise studies clustering at the top center and smaller, less precise studies spreading out at the bottom. In these funnel plots where three dots are close to the center line and exhibit small variances, it suggests that these studies, likely larger or more precise due to their small variances, are closely aligned with the overall effect size estimated by the meta-analysis. This alignment indicates consistency among these studies and contributes to the robustness of the meta-analysis findings. The positioning of these dots does not immediately suggest publication bias.

All three included studies achieved a NOS score of 7 or higher, demonstrating a robust study design and a minimized risk of introducing bias into our meta-analysis results (Table 3). In funnel plots, a nearly symmetrical distribution of studies around the mean effect size indicates that studies with both high and low statistical power are equally likely to be published, regardless of their findings, thus suggesting a low risk of publication bias and supporting the reliability of the overall meta-analysis results (Figure 3). In sensitivity analysis, adding or removing any of the three cohorts did not alter the significance of the results.

### 3.4. Functional Relevance of the LOXL1 Coding Variants

We checked the functional relevance of the three *LOXL1* gene SNPs (rs1048661, rs3825942, rs2165241) (Table 4). SNPs rs1048661 and rs3825942, located in Exon 1, result in Arg141Leu and Gly153Asp amino acid changes, respectively, while rs2165241 is situated in intron 1. SIFT predictions suggest deleterious effects for rs1048661 and rs3825942, conflicting with benign classifications by PolyPhen, pointing to the need for further functional assays. High CADD scores for rs1048661 (24.4) and rs3825942 (22.9) suggest significant deleterious potential, supported by RegulomeDB scores indicating regulatory roles. eQTL analysis shows tissue-specific expression variations: rs1048661 and rs3825942 in cultured fibroblasts (*p*-values: 1.40 × 10^−12^, 8.50 × 10^−37^) and rs2165241 in the pituitary gland (*p*-value: 1.30 × 10^−10^).

### 3.5. Power Analysis

In an association study, with 90 participants in the treatment group and 300 in the control group, the study would achieve a 39% statistical power to detect an odds ratio of 0.75 for group proportions. The allele frequencies were expected to be 0.5 under the null hypothesis (H0) and 0.43 under H1 in cases, while remaining constant at 0.50 in the control group. A two-sided Z-test with pooled variance was utilized, targeting a significance level of 0.05. It is important to note that for SNPs with allele frequencies less than 50% and an anticipated OR between 0.75 and 1.33, the statistical power to detect a difference between cases and controls at a significance level of 0.05 will be lower than 39%, suggesting the existing *LOXL1* association studies are all underpowered. In addition, our meta-analyses, after combining all available data, achieved a statistical power of 37% for rs1048661, 34% for rs3825942, and 5% for rs2165241.

Our power analysis results revealed that the insignificant findings in the meta-analysis at the conventional threshold of *p* < 0.05 could be due to the underpoweredness of the tests.

## 4. Discussion

In this study, we explored the intricate relationship between PDS/PG and their phenotypic and genetic overlap with XFS/XFG. Our systematic literature review and subsequent meta-analysis focused on the role of *LOXL1* gene variants in PDS/PG, identifying three genetic association studies that examined 10 *LOXL1* variants. Of these, two nonsynonymous coding variants, rs1048661 and rs3825942, showed nominal significance (*p* ≤ 0.01) in their association with PDS/PG, with no heterogeneity detected (I^2^ = 0). However, a power analysis revealed limitations in statistical robustness, both in the published studies and in our pooled analysis, indicating a need for larger sample sizes to detect significant differences. Our findings also highlight a gap in current research: key genes commonly associated with XFS/XFG, such as *CACNA1A*, *POMP*, *TMEM136*, *AGPAT1*, *RBMS3*, and *SEMA6A*, have not been adequately investigated in the context of PDS/PG. This underscores the necessity for comprehensive genetic studies, involving larger and more diverse cohorts, to fully determine the genetic underpinnings and distinctions between these ocular conditions.

The statistically insignificant findings in our meta-analysis should not be solely attributed to insufficient statistical power. There are other critical factors to consider, particularly in the clinical and genetic distinction between PDS/PG and XFS/XFG.

Despite the shared demographic and pathophysiological features between PDS/PG and XFS/XFG, key differences exist between these conditions (Table 5). Pigment dispersion syndrome/PG generally presents in younger individuals, predominantly males, and often has a genetic basis, with autosomal dominant or recessive inheritance patterns. This condition is marked by specific ocular features, including Krukenberg’s spindle, mid-peripheral ITD, and floating anterior chamber pigment, with the lens remaining normal. In contrast, XFS/XFG typically affects the elderly, shows a female predilection, and often follows an undefined inheritance pattern. Additionally, upon closer examination, there are subtle but crucial differences. For example, the anatomical locations of ITD in XFS/XFG (mid-peripheral iris) differ significantly from those in PDS/PG (pupillary border). The pattern and distribution of dispersed pigmentation in the two conditions also exhibit distinct characteristics (occasional endothelial pigment vs. Krukenberg’s spindle) [64]. These phenotypic variances are clinically significant and suggest underlying differences in their pathophysiology. Furthermore, the rarity of co-occurrence of XFS/XFG and PDS/PG in the same patients supports the hypothesis of their distinct nature. The singular case report by Pokrovskaya in 2016 illustrates this rarity and underscores the potential for distinct genetic underpinnings [64].

Our hypothesis posits that XFS/XFG and PDS/PG are genetically distinct entities, possibly sharing minor common genetic factors. This is supported by the phenotypic differences and the infrequency of their co-existence. Current genetic and genomic data on these conditions are not comprehensive enough to definitively map out the shared or differential genetic factors. Therefore, more extensive genetic and genomic research is necessary. Such research will not only clarify the genetic landscape of these conditions but also provide invaluable insights into their disease pathways.

The limitations of our study are primarily rooted in the scarcity and scale of available genetic data. The genetic landscape of PDS/PG is complex and not yet fully mapped, making the collection of comprehensive genetic information challenging. This inadequacy is further compounded by the limited sample size in our pooled analysis, which significantly undermined the statistical power of our meta-analysis. The inconsequential results, predominantly attributed to this small sample size, hinder our ability to draw robust conclusions about the genetic associations and differences between PDS/PG and other conditions, such as XFS/XFG. This paucity of data underscores the need for larger, more comprehensive studies that can provide a more accurate and holistic understanding of the genetic underpinnings of these ocular conditions. Consequently, future research endeavors should focus on expanding the genetic datasets with more substantial and diverse cohorts, which would enable more definitive conclusions and potentially reveal novel genetic insights into PDS and PG.

## 5. Conclusions and Future Directions

Our study suggests no association between *LOXL1* variants and PDS/PG. Further validation and exploration of XFS/XFG-associated genes in larger and more diverse cohorts would be helpful to determine the genetic correlation or distinctiveness between these conditions.

## Figures and Tables

**Table 1:** Summary of studies that tested *LOXL1* SNPs in pigment dispersion syndrome/pigmentary glaucoma.

Author	Country	Ethnicity	Phenotypes	Sample Size		Age of Cases/Control	Studied SNPs	Ref.
				Case	Control			
Giardina, E., et al. (2014)	Italy	Caucasians	PDS/PG	84	200	48.0 ± 4.9 / 46.7 ± 5.2	rs1048661, rs3825942, rs2165241, rs2304722, rs8818, rs3522	[39]
Wolf, C., et al. (2010)	German	Caucasians	PG	88	280	53.8 ± 13.5 / 66 ± 13	rs2165241, rs1048661, rs3825942, rs893818, rs1530169, rs750460, rs1284049	[38]
Rao, K.N., et al. (2008)	USA	Caucasians	PDS+PG	78	108	Not reported	rs1048661, rs3825942, rs2165241	[37]

PDS, pigment dispersion syndrome; PG, pigmentary glaucoma; SNP, single nucleotide polymorphisms.

**Table 2:** Data extracted for meta-analysis.

SNP	Position (GRCh38)	Functional Relevance	Author (Year)	Phenotypes Studied	Sample Size		Effect Allele	Ref. Allele	A1 Frequency	
					Case	Control			Cases	Controls
rs1048661	Chr15:73927205	Exon 1 Arg141Leu	[39]	PDS/PG	84	200	T	G	0.340	0.290
			[38]	PG	88	280	T	G	0.369	0.340
			[37]	PDS + PG	78	108	T	G	0.326	0.276
rs3825942	Chr15:73927241	Exon 1 Gly153Asp	[39]	PDS/PG	84	200	A	G	0.160	0.200
			[38]	PG	88	280	A	G	0.119	0.154
			[37]	PDS + PG	78	108	A	G	0.148	0.178
rs2165241	Chr15:73929861	Intron 1	[39]	PDS/PG	84	200	T	C	0.490	0.500
			[38]	PG	88	280	T	C	0.488	0.491
			[37]	PDS + PG	78	108	T	C	0.514	0.471

**Table 3:** Quality assessments of included case-control studies.

Author (Year of Publication)	Newcastle–Ottawa Quality Assessment Scale for Case-Control Studies									Reference
	Selection				Comparability	Exposure			Total Stars	
	1	2	3	4	1	1	2	3		
Giardina, E., et al. (2014)	a	a	b	a	age, sex, ethnicity	a	a	a	8	[39]
Wolf, C., et al. (2010)	a	a	b	a	age, sex, ethnicity	a	a	a	8	[38]
Rao, K.N., et al. (2008)	a	a	b	a	not reported	a	a	a	7	[37]

**Table 4:** Functional relevance of *LOXL1* variants.

SNP	Position	Gene	Location	SIFT	PolyPhen	CADD	RegulomeDB *	eQTL Tissue (*p*)—Allele for Higher Expression
rs1048661	Chr15:73927205	*LOXL1*	Exon 1 Arg141Leu	Deleterious	Benign	24.4	1f	Cells—Cultured fibroblasts (1.40 × 10^−12^)—G
rs3825942	Chr15:73927241	*LOXL1*	Exon 1 Gly153Asp	Deleterious	Benign	22.9	1b	Cells—Cultured fibroblasts (8.50 × 10^−37^)—A
rs2165241	Chr15:73929861	*LOXL1*	Intron 1	na	na	9.749	1f	Pituitary (1.30 × 10^−10^)—C

* 1b, eQTL/caQTL + TF binding + any motif + footprint + chromatin accessibility peak; 1f, eQTL/caQTL + TF binding/chromatin accessibility peak. The complete scoring scheme is available at “https://www.regulomedb.org/regulome-help/”. CADD, combined annotation-dependent depletion score; eQTL, expression quantitative trait locus; na, not available; PolyPhen, polymorphism phenotyping score; RegulomeDB, link: https://cherrylab.stanford.edu/projects/regulomedb; SIFT, sorting intolerant from tolerant score.

**Table 5:** Phenotypic comparisons between PDS/PG and XFS/XFG.

	PDS/PG	XFS/XFG
**Comparable manifestations**		
Prevalence	More common in Caucasians	More common in Caucasians
Cornea	Krukenberg’s spindle	Occasional endothelial pigment
Iris	Mid-peripheral iris transillumination defects	Transillumination at pupillary border
Iridocorneal angle	Open, posterior iris bowing, TM pigmentation	Open, TM pigmentation
**Different manifestations**		
Age of onset	Young	Elderly
Gender preference	Male	Female
Inheritance	Autosomal dominant, autosomal recessive, or sporadic	Undefined
Anterior chamber	Floating pigment	Quiet
Exfoliation material	None	Visible on pupillary border and anterior lens
Lens	Normal	Exfoliation material, weak zonules

PDS, pigment dispersion syndrome; PG, pigmentary glaucoma; XFS, exfoliation syndrome; XFG, exfoliation glaucoma.

## Appendix A. Newcastle–Ottawa Quality Assessment Scale for Case Control Studies

NEWCASTLE—OTTAWA QUALITY ASSESSMENT SCALE
CASE CONTROL STUDIES
(accessed via http://www.ohri.ca/programs/clinical_epidemiology/oxford.asp)
Note: A study can be awarded a maximum of one star for each numbered item within the Selection and Exposure categories. A maximum of two stars can be given for Comparability.
Selection

(1) Is the case definition adequate?
  (a) Yes, with independent validation.
  (b) Yes, e.g., record linkage or based on self-reports.
  (c) No description.
(2) Representativeness of the cases
  (a) Consecutive or obviously representative series of cases.
  (b) Potential for selection biases or not stated.
(3) Selection of Controls
  (a) Community controls.
  (b) Hospital controls.
  (c) No description.
(4) Definition of controls
  (a) No history of disease (endpoint).
  (b) No description of source.

Comparability

(1) Comparability of cases and controls on the basis of the design or analysis
  (a) Study controls for _______________ (select the most important factor: ethnicity).
  (b) Study controls for any additional factor (these criteria could be modified to indicate specific control for a second important factor).

Exposure

(1) Ascertainment of exposure
  (a) Secure record (e.g., surgical records).
  (b) Structured interview where blind to case/control status.
  (c) Interview not blinded to case/control status.
  (d) Written self-report or medical record only.
  (e) No description.
(2) Same method of ascertainment for cases and controls
  (a) Yes.
  (b) No.
(3) Non-response rate
  (a) Same rate for both groups.
  (b) Non respondents described.
  (c) Rate different and no designation.
